# SHAPE Asia: a regional community of practice for transforming food environments

**DOI:** 10.1080/16549716.2026.2684776

**Published:** 2026-06-25

**Authors:** Elaine Q. Borazon, Sirinya Phulkerd, Avita A. Usfar, Mohd Jamil Sameeha, Nisha Arunatilake, Anne Marie Thow, Bee Koon Poh

**Affiliations:** a IGPEHD, College of Social Sciences, National Sun Yat-sen University, Kaohsiung, Taiwan; b Institute for Population and Social Research, Mahidol University, Nakhon Pathom, Thailand; c Reconstra Utama Integra, Jakarta, Indonesia; d Centre for Community Health Studies (ReaCH), Faculty of Health Sciences, Universiti Kebangsaan Malaysia, Kuala Lumpur, Malaysia; e Institute of Policy Studies of Sri Lanka, Colombo, Sri Lanka; f Leeder Centre for Health Policy, Economics and Data, Faculty of Medicine and Health, School of Public Health, The University of Sydney, Camperdown, Australia

**Keywords:** Public health, policy, coalition, collaborative learning network, Asia

## Abstract

Rapidly changing Asian food environments characterized by increased availability of cheaper, energy-dense, and low-nutrient products, are negatively impacting nutritional and health outcomes. This paper introduces SHAPE Asia, a collaborative learning network of food environment interest-holders across Indonesia, Malaysia, Thailand, the Philippines, and Sri Lanka. These countries face significant regulatory challenges. Unregulated food marketing and retail practices promote unhealthy products resulting in adverse health impacts. Policymaking is hampered by insufficient political will, industry interference, and government sensitivity to perceived economic impacts of regulation compounded by inadequate public awareness and limited prioritization of sustained behavior change. Balancing economic and public health interests requires multifaceted, multisectoral approaches to ensure commitment toward healthier food environments. SHAPE Asia generates regionally-specific knowledge through shared learning to support double-duty food policy actions. It engages policymakers, academics, government agencies, international organizations, media, and civic organizations to ensure inclusive and equitable food environment transformation. It facilitates peer learning and South-South and North-South capacity building supporting healthy food environment advocacy. It translates research into action by strengthening coordinated efforts to develop feasible, effective, and sustainable policy options while building interest-holder support and generating policy-relevant evidence. SHAPE Asia is part of a global cohort of multi-actor coalitions influencing policies and transforming food systems across Central and South America, South Asia, and Southeast Asia. These coalitions combine collaborative learning networks and communities of practice to support production, consumption, and access to healthy diets, particularly for vulnerable populations, while emphasizing sustainable, gender-equitable, and inclusive systems contributing to the health of people and ecosystems.

## Paper Context

- **Added knowledge:** This paper provides the first comprehensive documentation of how South-South and North-South collaborative learning networks can generate regionally-specific, actionable knowledge to support double-duty food policy development while building multi-sectoral interest-holder capacity for sustainable food environment transformation.
- **Global health impact for policy and action:** SHAPE Asia offers a replicable model for coordinating multi-actor coalitions to translate research into feasible policy options that balance economic and public health interests, with potential application to other regions experiencing rapid nutrition transitions and weak regulatory frameworks for unhealthy food environments.

## Background

Over the past several decades, commercialization of food retail and marketing in Asia has profoundly reshaped food environments, social norms, and dietary behaviors [1–3]. The rapid expansion of modern retail outlets, aggressive marketing of ultra-processed and energy-dense foods, and normalization of convenience-driven consumption have shifted diets away from traditional, minimally processed foods toward unhealthy dietary patterns [4,5]. These changes are now a major driver of rising malnutrition in all its forms and diet-related non-communicable diseases (NCDs) across the region [4,6].

The increasing prevalence of malnutrition and non-communicable diseases (NCDs) in Asia over recent years has been alarming. Despite modest progress in reducing stunting (from 108.8 million to 76.8 million) and wasting (from 37.1 million to 30.0 million) [7], adult obesity nearly doubled from 192.9 million to 353.9 million between 2012 to 2024 [4], with overweight and obesity cases projected to worsen by 2030, especially in Eastern and Southeastern Asia [7]. Undernutrition remains heavily concentrated in Asia, which in 2024 accounted for approximately 323 million undernourished people, nearly half the global total, despite a substantial decline from 570 million in 2004 [4]. At the same time, poor diets high in energy-dense, nutrient-poor foods have driven diet-related NCDs across LMICs, accounting for nearly two-thirds of deaths in Southeast Asia [8].

Urbanization, rising incomes, and lifestyle changes have driven significant shifts in food demand and consumption patterns across Asia, with nutrition transition [9] predominantly occurring in urban areas [10] due to increased availability of processed foods in the market [11] and emergence of global supermarkets and fast-food chains that have contributed to westernized diets characterized by higher consumption of energy-dense, nutrient-poor products [12]. Food marketing and retail environments increasingly influence what foods are visible, affordable, and socially desirable, shaping eating habits and reinforcing unhealthy norms over time [13,14].

While high-income countries have begun implementing stricter regulations on food marketing and retail to protect children [15], many Asian countries face regulatory gaps and limited enforcement capacity [16,17]. Although such regional initiatives as the ASEAN minimum standards on protecting children from harmful food marketing represent important progress [18], policy responses across Asia remain fragmented and insufficiently informed by region-specific evidence, hampering progress toward global nutrition targets [19]. With Asia’s urban population projected to increase by 83% by 2050, and in South Asia by 120% [20], shaping healthy food environments is critical for enabling better dietary choices and nutritional outcomes [21,22], yet research translation into actionable policies has been limited, constraining governments’ ability to effectively regulate food environments [23,24].

Addressing these challenges requires coordinated, research-driven, and context-specific action across countries. Asia’s diversity in food cultures, governance systems, and market structures means that solutions must be adapted to local contexts while benefiting from shared learning and regional cooperation. Evidence shows that there is significant potential for the region to achieve ‘mutual learning’ through the cooperation of all countries [25]. International joint movements and policy harmonization across countries are effective means of addressing transboundary issues [26], such as food systems, nutrition and health, in Asia, particularly by reinforcing governance solutions. Effective policy harmonization and international coordination are demonstrated through several initiatives in other regions that manage transboundary food system challenges. For instance, European Commission’s common definition of food waste enabled integration of diverse national legislations and improved synergy across EU food safety and security policies [26], while Codex Alimentarius Commission (FAO/WHO) provides internationally harmonized food safety standards that serve as the global reference for settling trade disputes through the World Trade Organization [27]. The strengths in diversity of food cultures, expertise, and practices can be capitalized to develop long-term and impactful changes in the community. With more than half of the world’s population situated in Asia, the collective influence of a regional alliance will be more effective in amplifying public health challenges in global policy discussions.

Translating this regional potential into coordinated action depends on a robust evidence base, yet food environment research in Asia remains fragmented and unsystematized, with limited coordination mechanisms among interest-holders, groups with a legitimate stake in the health issue at hand [28], to consolidate findings and translate them into context-specific policy [24,29]. Strengthening this research infrastructure is therefore foundational to the regional cooperation outlined above, without which harmonized policy responses to Asia’s nutrition challenges will remain out of reach.

In recognition of the urgent need to transform food environments in Asia through adequate research, policy monitoring and implementation, and policy advancements, a strong network or community of practice is necessary to mobilize support from interest-holders including government, non-government organizations, advocates, and community leaders. A community of practice (CoP) is defined as a group of people sharing a concern, domain of knowledge, and practice, who learn from each other through regular interaction and collaboration [30]. Comparable regional CoPs include Africa’s Food Environment Research Network (FERN), which has successfully identified priority areas for food environment research and policy in Africa [31,32], and *Comunidad de Práctica Latinoamérica y Caribe Nutrición y Salud* (COLANSA) in Latin America and the Caribbean, which has strengthened regional capacity and generated evidence to support public health policies on food environments and NCD prevention [33].

In response to the commercialization-driven transformation of food environments and the lack of coordinated policy action in Asia, SHAPE Asia (*Shaping Healthier Food Systems and Policy Environment in Asia*), was established as a regional community of practice and collaborative learning network modeled on similar initiatives in Africa (FERN) and Latin America (COLANSA). Although SHAPE Asia operates independently of FERN and COLANSA, and is not institutionally affiliated with them, the two initiatives share a common community-of-practice model, such as convening researchers, policymakers, civil society actors, and advocates, around a shared agenda of food environment transformation. SHAPE Asia aims to strengthen evidence generation, foster cross-country learning, and support advocacy for healthier and more equitable food environments across Indonesia, Malaysia, Thailand, the Philippines, and Sri Lanka. As a CoP, it provides a sustained platform for collective learning, joint problem-solving, and coordinated action across member countries, distinguishing it from time-bound research consortia or one-off policy dialogues. While the network initially focuses on food retail and food marketing building on the foundational work of the South East Asia Obesogenic Food Environment (SEAOFE) study, which examined retail food environments, consumer and retailer perspectives, and existing food retail policies to develop evidence-informed policy recommendations [34], this serves as a strategic entry point rather than a limitation. SHAPE Asia is designed to evolve and expand into other critical food environment domains, such as food promotion, provision, labeling, pricing, and trade, based on emerging policy opportunities and country-specific priorities.

By integrating research, policy engagement, and coalition-building through its community-of-practice architecture, SHAPE Asia provides a structured and context-sensitive solution to counter the negative impacts of unregulated food marketing and retail, contributing to improved diets, reduced NCD burdens, and progress toward the Sustainable Development Goals, particularly SDG 2, SDG 3, and SDG 17.

## The SHAPE-Asia network: objectives and approach

The foundations of SHAPE Asia were laid through the SEAOFE project, the first regional effort to examine food environments in a structured and comparative way [34]. Through policy analysis, community assessments, and interest-holder engagement, SEAOFE generated core evidence on drivers of unhealthy diets and established the collaborative foundation [34] on which SHAPE Asia now builds. While SEAOFE is a time-bound research study focused on retail food environments in four Southeast Asian countries, SHAPE Asia is a sustained community of practice encompassing advocacy, policy engagement, capacity strengthening, and implementation support across the broader food environment domains and an expanding geographic footprint that now includes Sri Lanka.

The co-creation of SHAPE Asia built on this foundation, together with earlier collaboration with the South Asia group, linked through key officers from the International Development Research Centre (IDRC), Canada, which provided funding support for SEAOFE. These parallel efforts helped establish trust, strengthen regional connections, and align approaches, providing the platform for SHAPE Asia to emerge as a broader coalition connecting partners from Southeast and South Asia around shared learning, collective action, and policy-focused efforts to improve food environments.

Given this foundation, SHAPE Asia operates as a collaborative learning network addressing food environment challenges through an evidence-informed research agenda, policy engagement, and capacity building, centered on four interconnected objectives: (i) developing strategic partnerships to drive change toward healthy, gender-equitable, and sustainable food environments; (ii) generating an evidence base through shared learning to support double-duty food policy action; (iii) influencing policy change by mobilizing interest-holders and collaborating with policymakers; and (iv) improving policies through effective knowledge communication and advocacy.

SHAPE Asia is anchored by academic researchers and public health scholars from universities and research institutions across Indonesia, Malaysia, Thailand, the Philippines, and Sri Lanka, and is guided by a board of advisors with expertise spanning food environment research, public health, nutrition, policy analysis, and gender studies. Leadership and coordination of SHAPE Asia is provided by the core group of convenors (researchers from Indonesia, Malaysia, Thailand, the Philippines, and Sri Lanka) who lead national-level activities in their respective countries and ensure country-specific priorities are reflected in the network’s strategic directions. These five countries were selected on two grounds: Indonesia, Malaysia, Thailand, and the Philippines were part of the SEAOFE team and therefore brought established research foundations, country-level partnerships, and shared methodologies into the network, while Sri Lanka was incorporated as an entry point for progressive expansion into South Asia. While the network maintains a strong research orientation, its members occupy multiple roles as policy advisors, capacity builders, and advocates working alongside government agencies and civil society organizations.

SHAPE Asia engages the wider research, civil society, and advocacy community through policy labs and dialogues that serve both as dissemination channels and platforms for new partner engagement. Participation is open to researchers, civil society organizations, advocates, and policy actors working on food environments in Asia, with membership guided by relevance of interest and influence and by conflict-of-interest safeguards. The network operates across multiple scales from local community initiatives to national policy advocacy to regional knowledge exchange, recognizing that food environment challenges require coordinated action at each level.

SHAPE Asia explicitly recognizes the critical role of women and marginalized groups in sustaining healthier food environments. While all genders shape dietary patterns, women typically serve as primary household decision-makers regarding food purchase, selection, and preparation. The network advances gender-transformative initiatives that increase health and nutrition literacy across all genders while ensuring equitable resource allocation and decision-making power. For example, SHAPE Asia could in the future co-design food environment initiatives with women and girls, support women- and youth-led activities promoting healthy food environments, and advocate for local food-environment regulations that require gender-balanced representation in food-policy task forces. Within SHAPE Asia, we work towards balanced gender representation across all internal structures and partner initiatives, and we advocate for governments to institutionalize gender equity in food system governance to ensure diverse groups can meaningfully contribute to decision-making processes.

SHAPE Asia acknowledges the considerable power of food industries to hinder policies promoting healthier food choices. To mitigate undue influence, all members are required to disclose connections with food industry actors, with partner initiatives expected to adopt similar safeguards. Consistent with WHO guidance [35,36], we distinguish between legitimate private sector participation in policy implementation and direct involvement in policy formulation, where structural conflicts of interest may compromise public health objectives [37].

## SHAPE Asia’s theory of change

**Figure 1.**
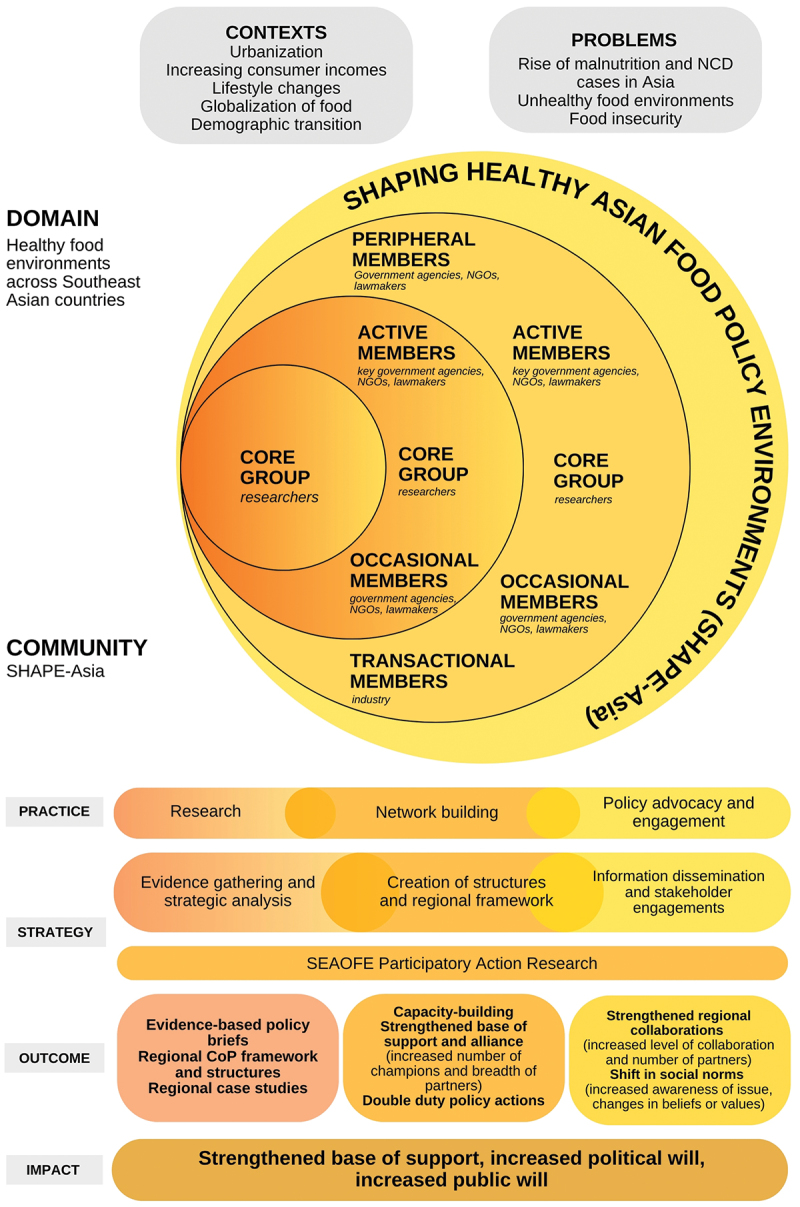
SHAPE Asia’s theory of change framework.

SHAPE Asia’s theory of change is anchored in the Advocacy Coalition Framework (ACF) proposed by Sabatier and Jenkins-Smith [38], built around five outcomes, namely strengthened base of support, shifts in social norms, strengthened alliances, improved policies, and impact (changes in social and/or physical conditions), while incorporating the elements (domain, practice, community) of a CoP. The theory articulates how the formation of a community of practice can lead to changes in policy outcomes through its network of actors, shared beliefs and interests, and group dynamics [39] by focusing on knowledge sharing and collaborative learning. A comprehensive framework of the theory of change is presented in Figure 1, which illustrates the contexts and problems alongside proposed practices and strategies to achieve the target outcomes and impacts of the network.

The ACF suggests that the policy subsystem is key to understanding policy processes. Policy subsystems define the topic or domain of policies and the potential actors involved in shaping policy outcomes [40]. In the proposed theory of change (see Figure 1), food environments are identified as the policy subsystem whose components, including food composition, labeling, promotion, provision, retail, prices, and trade and investment [41], constitute the target domain for coordinated policy action. SHAPE Asia will initially focus on food retail and marketing and will scale up contingent on government interests. SHAPE Asia will operate through three interconnected strategies: (1) Creation of Structures and Regional Frameworks, (2) Evidence Gathering and Strategic Analysis, and (3) Advocacy and Scaling. These strategies are integrated to promote synergistic efforts across SHAPE Asia’s initiatives and are designed to be implemented over an initial two-year establishment phase, with Strategy 1 laying structural foundations, Strategy 2 generating evidence through participatory action research, and Strategy 3 scaling advocacy as the coalition matures. Activities will continue beyond this period subject to funding cycles, consistent with the sustained community-of-practice model underpinning the network. The conceptual basis and operational implementation of each strategy are presented together in Section 4. Specific tools, instruments, and procedures for each strategy are documented in dedicated project protocols and forthcoming network publications.

Within this framework, SHAPE Asia categorizes interest-holders into four engagement tiers based on their level of interest and influence in food environment policy: active members (high-impact actors who participate regularly), occasional members (key actors involved only in specific discussions, peripheral members (actors with minimal involvement), and transactional members (actors engaged for specific benefits, such as industry). Engagement strategies are tailored to each tier and engagement expands progressively across the three strategies as the network matures.

Ultimately, SHAPE Asia’s activities are directed toward strengthening political and public will for healthier food environment policies, with anticipated outcomes and impacts detailed in Figure 1. This theory of change underscores the network’s comprehensive approach to transforming food policy landscapes through strategic research, interest-holder collaboration, and continuous advocacy.

## Operationalization of SHAPE Asia

SHAPE Asia operationalizes its Theory of Change through coordinated country-level and regional actions that translate shared learning, evidence generation, and advocacy into concrete policy-relevant outputs. Building on the foundation of the SEAOFE research group, SHAPE Asia initially concentrates on food retail and food marketing across five countries, namely Indonesia, Malaysia, Thailand, the Philippines, and Sri Lanka, while establishing mechanisms for regional coordination, peer learning, and scaling.

This section presents each of SHAPE Asia’s three strategies in turn, integrating its conceptual grounding within the ACF with concrete activities through which it is being implemented.

### Strategy 1: creation of structures and regional frameworks

Grounded in the ACF’s focus on the policy subsystem and its actors [38], the first strategy focuses on creating structures and regional frameworks for SHAPE Asia centered on food retail and food marketing within five focus countries, using existing policy mapping data from the SEAOFE food retail study [42]. National and regional advisory groups will be created to guide SHAPE Asia’s strategic directions, support implementation, and ensure contextual relevance. National advisory groups provide country-specific insights into political, institutional, and cultural contexts, while a regional advisory group facilitates alignment, cross-country learning, and coherence across SHAPE Asia activities. Members are selected based on demonstrated expertise in food environments or public health nutrition, active engagement with relevant policy processes, gender balance, and absence of conflicts of interest with commercial food and beverage actors.

Concurrently, interest-holder mapping will be conducted to categorize actors into active, peripheral, occasional, and transactional members based on levels of interest and influence, with engagement strategies tailored to each category. This process supports effective collaboration by clarifying the roles of active, occasional, peripheral, and transactional actors across research, advocacy, and dissemination activities.

At the regional level, SHAPE Asia developed a shared operational framework for food retail and food marketing to support harmonized analysis across countries. This framework was informed by a scoping review of existing food environment frameworks [18,22,41] and refined through consultative meetings held between August 2025 to January 2026, with regional interest-holders. The framework serves as a practical tool to guide joint analysis, identify priority gaps, and support coordinated action across national contexts.

### Strategy 2: evidence gathering and strategic analysis

Drawing on PAR methodology [43] and the concept of policy-oriented learning within the ACF [38], the second strategy involves evidence gathering and strategic analysis to inform policy interventions through collective action to identify interest-holder types, build frameworks for identifying policy intervention points, assess policy and intervention maturity and gaps, and actively involve interest-holders throughout the research process to ensure findings are actionable and relevant. Gender-disaggregated data will serve as the empirical basis for policy recommendations and advocacy for healthy food retail and marketing practices.

Evidence generation is implemented through country-led research activities, including evidence synthesis, policy mapping, and data collection, supported by regional methodological alignment to ensure comparability and shared learning. Core group members from all five countries undergo capacity building in research methods and policy-oriented learning [44] to generate evidence-based knowledge products and strengthen their collective role in network governance and decision-making.

A PAR approach is used to bridge research and practice. PAR enables interest-holders, including researchers, policymakers, and civil society actors, to jointly identify problems, test policy-relevant interventions, and reflect on outcomes. This approach strengthens policy relevance and supports policy-oriented learning over time.

Capacity building activities are delivered regionally and applied nationally to equip core group members with skills in research design, policy analysis, and evidence translation. These activities strengthen the collective capacity of SHAPE Asia while allowing countries to adapt methods to their specific institutional and political contexts.

At this stage, engagement focuses on active and occasional members, whose institutional and technical input is central to evidence generation. The main outcomes of network building at this stage are to capacitate more members, strengthen the base of the alliance, and draft double-duty policy action proposals.

### Strategy 3: advocacy and scaling

Grounded in knowledge-brokering and knowledge-translation frameworks [45,46] and informed by precedents from Latin American front-of-pack labelling coalitions [47] and African food environment networks [32], the third strategy focuses on advocating for policy change and effective implementation through awareness-raising and enabling (through training and evidence) and mobilizing interest-holders. SHAPE Asia will draw its strength by forming a network of knowledge brokers with diverse expertise to achieve a common interest in healthier food environments. This strategy will influence policies through strategic evidence creation, knowledge translation, and the creation of supportive networks. SHAPE Asia will use a variety of methods, including policy briefs, advocacy briefs, on-line and off-line policy dialogues, one-to-one meetings, to raise awareness and influence policies using strategic platforms and policy windows. Equity and gender considerations are embedded in SHAPE Asia’s advocacy approach: policy dialogues and forums are designed to ensure inclusive participation across genders and marginalized groups, and knowledge products explicitly address how food environment policies can advance gender-equitable outcomes.

The core group will spearhead the expansion and network-building process based on emerging opportunities for food environment policy improvement. They will also identify key interest-holders in their respective countries who will participate in knowledge sharing, workshops, training, forums, and think tank sessions. SHAPE Asia will facilitate knowledge sharing dimensions through formal and informal approaches. Formal approaches include regular brainstorming sessions, meetings, and workshops, the creation of central knowledge repositories, and mentorship programs. Informal approaches include sharing of personal experiences, online discussions and forums, and social events and networking.

In addition to active and occasional members, the network will further expand and communicate with potential peripheral and transactional members. A participatory approach through open invitations will be used to engage potential members into the community of practice. Beyond the iron triangle – legislation, interest groups, and government agencies – other identified actors include researchers, non-government organizations, academia, consumer groups, and industry. The involvement of the industry will be limited to the final stage during information dissemination to increase awareness and knowledge towards healthier food environments and policies. These engagements will be carefully designed and managed to avoid the influence of conflicting interests on policies through public webinars and interest-holder forums which aim to expand SHAPE Asia membership and to strengthen regional collaboration.

This engagement model safeguards evidence generation and policy formulation from commercial conflicts of interest, consistent with WHO guidance [36,48], while providing industry a clearly defined role in implementation and awareness-raising. Its limitation is that it foregoes market-level knowledge that industry could contribute to policy feasibility, and assumes industry will engage constructively with recommendations developed without their early input, and these may not hold uniformly across contexts [49].

Advocacy efforts focus on setting research agenda that inform and influence policy, conducting research needed for filling gaps necessary for policy formulation and awareness building, and creating policy-relevant outputs at national and regional level. In order to showcase co-creation, country teams engage with policy makers for identifying research agendas, prioritizing advocacy activities, abuilding awareness and capacities, and supporting with policy formulation.

SHAPE Asia contributes to policy implementation gap through generating and monitoring evidence that enables governments and civil society to track industry compliance with existing regulations, supporting development of clearer implementation guidelines, compliance indicators, and accountability mechanisms that translate policy text into enforceable practice, and strengthening national coalitions to hold regulators and industry accountable through transparent reporting. While SHAPE Asia is not a regulatory body and does not enforce compliance directly, it builds the evidence and coalition infrastructure that enables national authorities to do so more effectively.

The food and beverage industry sector is expected to adhere to government-formulated regulations through product reformulation, improvements in promotion of healthier products, and participation in compliance monitoring and transparent reporting. We recognize that these changes are more likely to be achieved through a combination of regulatory pressure, market incentives, and shifting social norms than through awareness-raising alone, and SHAPE Asia’s advocacy, evidence generation, and coalition-building activities are designed to strengthen all three drivers in parallel.

## Network expansion and long-term vision

Network expansion strategies include establishing partnerships with high-interest, high-influence interest-holders identified through interest-holder mapping under strategy 2; developing partnerships with researchers and government bodies in South Asian and other Southeast Asian countries; and exploring opportunities in food labeling policies, where government interest and regulatory momentum create entry points for network engagement while remaining responsive to government interest and regulatory opportunities arising during interest-holder engagements. These activities build on initial foundations to support informed consumer choices and healthier food environments.

## Long-term vision and impact

SHAPE Asia aims to establish itself as the leading regional network in Asia for fostering collaboration, cross-learning, and policy influence related to creation of healthier food environment. Initial focus on Southeast Asia will expand progressively to South Asia and other Asian countries. Through sustained evidence-based research, robust regional framework development, policy learning, and strategic advocacy, SHAPE Asia will empower local and regional communities, promote equity and gender inclusivity, and ensure healthier food systems.

SHAPE Asia’s long-term ambition extends beyond policy adoption to effective translation into practice, supporting national actors in closing implementation gaps through monitoring, compliance indicators, capacity strengthening, and iterative policy refinement.

This long-term direction is informed by concrete lessons from food environment coalitions in other regions. The Latin American experience demonstrates that sustained civil society advocacy grounded in independent evidence is essential for advancing contested nutrition policies [47], while African food environment coalitions illustrate the value of embedding regional coordination within existing policy architectures [32], which has informed SHAPE Asia’s alignment with ASEAN platforms and national NCD frameworks. A cross-cutting lesson is that networks investing early in monitoring infrastructure and accountability mechanisms sustain impact over time.

Finally, these efforts contribute to achieving Sustainable Development Goals 2 (Zero Hunger), 3 (Good Health and Well-being), and 17 (Partnerships for the Goals), advancing healthier and more resilient food systems across the Asian region.

## Data Availability

Data sharing is not applicable to this article as no data were created or analyzed in this study.
